# Factors Predicting Physical Activity and Sports Participation in Adolescence

**DOI:** 10.1155/2021/9105953

**Published:** 2021-02-24

**Authors:** Amalie Rullestad, Eivind Meland, Thomas Mildestvedt

**Affiliations:** Department of Global Public Health and Primary Care, University of Bergen, Bergen, Norway

## Abstract

Physical activity is important for children's health and wellbeing, yet participation declines across teenage years. It is important to understand the mechanisms that could support adolescents to maintain physical activity participation. The aim of this study was firstly to examine change in sports and nonsports activities over two years during adolescence. Secondly, we explored possible predictors of physical activity and sports participation after two years. *Method*. A longitudinal cohort study was conducted between 2011 and 2013. Our data were collected from 1225 Norwegian adolescents who were followed over a two-year period, from 6th to 8th grade (11 to 13 years) and from 8th to 10th grade (13 to 15 years). We examined the relations between physical activity and predictors such as peer support, parent support, socioeconomic status (SES), attitude towards physical education, active transportation to school, self-rated health, body image, and change of nonsports activities. We used linear regression analyses and binary logistic regression to explore possible predictors of physical activity and sports participation after two years. *Results*. We found a significant reduction in sports participation during early adolescence, most pronounced, from 8th to 10th grade (from 13 to 15 years). Factors which predicted physical activity after two years were a positive attitude towards physical education, perceived support from parents, if the student travelled to school in an active way (by walk or bicycle) and also how the student rated his/her own health. The last three factors also predicted improvements of physical activity during the two years. Possible predictors of persisting or starting doing sports were increasing levels of self-rated health, increasing socioeconomic status, whereas increasing engagement in nonsports activities predicted reduced participation in sports. *Conclusion*. Health promotive efforts aiming at increasing active school transportation, parental support, and subjective health seem important for maintenance of physical activity and sports participation during adolescence. Attitudes may improve by adapting physical education to individual needs and interests and can function as an additional promotive factor.

## 1. Introduction

Physical activity (PA) and participation in sports are of great importance for children's health, and youths who engage in physical activity are more likely to be physically active as adults [1–3]. The physiological and psychological benefits of regular physical activity for children and adolescents are supported by a considerable literature [1, 4]. Being physically active is also considered as an important determinant when it comes to school performance [5]. Research shows that those who are well educated have better health and wellbeing, and there is increasing evidence that regular participation in physical activity is associated with enhancement of brain function and cognition. Thus, students in better health have higher academic attainment [6, 7].

While the benefits of PA are common knowledge, research suggests that among some populations and in some conditions, physical activity may be associated with negative consequences [8]. In a Norwegian cross-sectional study from 2014 investigating 2527 Norwegian adolescents aged 15–20, participation in sports with leanness advantage was associated with body dissatisfaction [9]. The study also revealed a strong association between time spent on physical activity and self-rated health (SRH), in a dose-response manner [9]. SRH is an individual's subjective perception of his or her own health status and it is an important predictor for later wellbeing and protects against diseases [10]. SRH is a relatively stable construct of self-identity during adolescence, although it is influenced by health-promotional factors as for instance PA [11].

According to the recommendations from the World Health Organization, children and adolescents should be active at least 60 minutes of moderate to high intensity every day [12]. They should also do activities with a high intensity at least three times a week, including activities that stimulate muscle growth and bone strength. Norway has launched similar guidelines [1].

Despite the many health benefits of PA, most adolescents do not reach the recommended levels of physical activity. In Norway, 87% of girls and 96% of boys participate in moderate PA for at least 60 min a day at the age of 6 years, but at the age of 15 years, only 43% of girls and 58% of boys reach this recommended level of PA [13]. 75% of teenagers between 13 and 18 years are participating in organized sports at some point, through their adolescence. At the same time, the dropout rate from youth sports is quite high. Almost six out of ten who have participated in youth sports have quit before they turn eighteen years old [14]. There is a precipitous decline in physical activity and participation in organized sports across the teenage years, highlighting the need to understand influences on PA and sports participation among young people [14–16]. Adolescence is a critical time to develop PA patterns which extend to adulthood [2, 3]. Therefore, identifying barriers and promotive factors of PA is important.

The recent Health Behaviour in School-Aged Children (HBSC) survey from the WHO reports that physical activity declines with age, particularly among boys. PA participation (both moderate-to-vigorous and vigorous physical activity) remains particularly low among girls and older adolescents. At all ages, boys are more likely to be physically active than girls, and PA is lower among older adolescents and those from low-affluence families [17].

The literature reveals several predictors for maintained physical activity in adolescence. In a Norwegian longitudinal study looking at factors predicting changes in PA through adolescence, 2348 adolescents aged >13 years were followed for approximately 4 years. The study revealed that predictors of change in or maintaining PA during adolescence differed by gender [18, 19]. Perceived overweight, dissatisfaction with life, and lack of active participation in sports at baseline were significant predictors for decreased PA among boys at follow-up. For girls, health-compromising habits such as tobacco and alcohol consumption, low maternal education, and maternal physical inactivity predicted relapsers (active but became inactive at follow-up) and inactive maintainers [18]. Higher levels of education and more physically active parents at baseline seemed to protect against decreased PA during follow-up for both genders [18]. Gender differences when it comes to predictors of PA were also found in an American longitudinal study from 2012. In this study, 578 adolescents, aged 10–16 at baseline, were followed for 2 years. The most powerful predictor of PA after 2 years, for both genders, was baseline levels of PA. PA at baseline is a consistent predictor for PA at follow-up in most studies [2, 18, 19]. For boys, greater self-efficacy and baseline moderate-to-vigorous physical activity (MVPA) was statistically associated with MVPA at follow-up. For girls, baseline MVPA and perceived barriers to PA significantly predicted MVPA at follow-up [19].

Previous research concerning predictors of participation or dropout in organized sports shows that differences in children's sports participation are best accounted for by sociocultural and socioeconomic indicators [20]. In an Australian longitudinal study from 2014, where they followed 4042 children from 8 to 10 years old, higher household income, higher parental education, parental support in sports activities, and access to a physical education (PE) teacher during primary school predicted sports participation [20]. In a longitudinal study from Denmark in 2011, they investigated the associations between sports participation and parental, social, and cultural factors in four Danish municipalities among 6356 Danish adolescents aged 12–16 years. Young age and male gender were associated with adolescents' sports participation. Adolescents were more likely to participate in sports if they perceived their parents as active in exercise or sports. The female adolescents were less likely to participate in sports with one or two unemployed parents, than adolescents with two employed parents [21]. A systematic review of social support in youth sport from 2013 found that coach, parent, and peer support plays a significant role in shaping youth sport experiences both from a positive (athlete motivation levels and elite sports participation) and negative (dropout) perspective [22].

Improving the PA levels of youth is an important public health challenge. Health promotion efforts should also consider the aspect of body dissatisfaction and body acceptance when promoting PA and sports [9]. Knowledge about patterns of participation can be used to identify activity promotive factors in order to guide public health efforts and design more effective interventions. Findings from previous research suggest that efforts to promote habitual daily physical activity by, for example, increasing opportunities for school-based activity, and active transportation and active leisure among adolescents can be of importance [17]. School PE is recognized as a key opportunity for improving PA amongst adolescents [23]. While there are several studies supporting a positive association between PA and SRH, these are mostly cross-sectional studies [9, 24]. In a longitudinal Norwegian study from 2009, following 2399 adolescents from 13 to 19 years, PA was a significant contributor to change in SRH over 4 years [11]. We have not found studies investigating if SRH is a predictor of future PA.

Most of the previously referred studies have used a cross-sectional design. Cross-sectional studies restrict the evidence to associated factors rather than to predictors (determinants) of PA [15]. Researchers in the field suggest that one explanation for the modest effect of existing interventions was that they have failed to adequately target the most important determinants of PA [25]. The literature revealing correlates and determinants of youth physical activity is namely inconsistent in terms of findings and methodological quality [25]. Therefore, the authors call for precise use of terminology and more studies with a longitudinal design which is more suited to assess causality, than cross-sectional models.

We have identified two other Norwegian studies with longitudinal cohort design with adolescents somewhat older than in the present study [18, 26]. The current study uses other predictors compared to the other longitudinal Norwegian studies, such as socioeconomic status (SES), attitudes towards PE, engagement in other leisure time activities, SRH, and body dissatisfaction. The present study is also a longitudinal cohort study and will complement the findings in earlier studies.

On this background, we firstly set out to examine change in sports and nonsports activities over two years during adolescence. Secondly, we explored possible predictors of physical activity and sports participation after two years. Finally, we conducted residual change analyses with the PA-status at T0 as an adjusting variable in order to explore predictors of change during the two years.

## 2. Material and Methods

**Figure 1:**
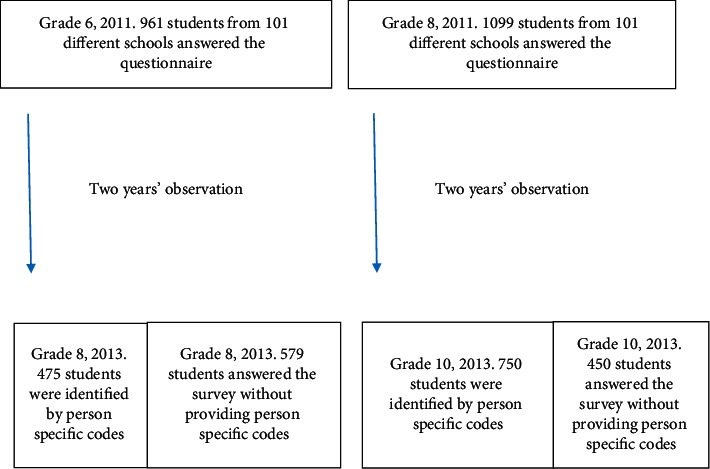
Students participating in the longitudinal cohort study from elementary and junior high schools in the former county of Sogn og Fjordane from 2011 to 2013. The longitudinal study was part of two cross-sectional studies in 2011 and 2013. Therefore, the total number of participants differs between the two years.

We invited all municipalities in the former county of Sogn og Fjordane in western Norway to participate in the survey, and all except one, accepted the invitation. Sixty-seven per cent of a total of 3075 students in grade 6 and grade 8 (2060 students) took part in 2011. In 2013, 72% of 4538 students from grades 6, 8, and 10 responded (2254 students from grades 8 and 10). One hundred and one different schools participated in both surveys. We have outlined the study design in Figure 1. The survey was administered late in the fall term (November/December) at both points in time. We considered this as a stable period in the semester, before testing and exams by the end of the fall term. Eighty-six per cent of the participants lived in rural municipalities. Public schools are attended by 97.8% of Norwegian students, and students are not normally organized according to level of ability, gender, or ethnic affiliation [27].

The main reason for nonparticipation was absence from school on the day of data collection. A few classes dropped out because of logistic problems, but the participation across grade levels was fairly identical: 1001 students in grade 6; 1054 in grade 8; and 1200 in grade 10. A great majority of students in grade 8 and 10 in 2013 answered the same survey in 2011, but we only identified 1225 by person specific codes across the two time-points due to insufficient coding. The coding insufficiencies were randomly distributed between persons and classes, although insufficiencies were more prevalent among the youngest (11 years in 2011). We followed a total of 612 boys and 613 girls: 475 from 6th to 8th grade and 750 from 8th to 10th grade, i.e., 1225 students with an almost identical sex distribution across the two cohorts (see Figure 1).

This cohort, surveyed and identified at both time-points, comprised 49% of the original students measured in grade 6 and 68% of the students measured in grade 8 in 2011. A vast majority of the students completed the questionnaires.

### 2.1. Measures

The questions regarding physical activity, self-rated health, and body dissatisfaction were based on the World Health Organization cross-national survey, Health Behaviour in School-Aged Children (HBSC), which aims to increase knowledge about health and lifestyle in adolescents [28]. These self-reported variables have proved reliable and valid among younger age groups [28, 29] and also among adolescents at similar age [11]. Internal consistency of composite variables was checked using Cronbach's alpha and was satisfactory as demonstrated in Table 1.

The outcomes consisted of two different measures. Physical activity was computed as the mean score of the two questions:

1. Outside school hours: how many days a week do you play sports or exercise so that you get out of breath or sweat?
2. During the last seven days, how many of these days have you been physically active for at least 60 minutes?

We computed the outcome called “persistent and started doing sports” based on the question: “how many days per week do you participate in organized leisure time activities and which activities?” Sports were one alternative, and they indicated how many days per week they did the activity. We recoded sports activity to a dichotomous variable, indicating if they participated in sports or not, and computed a new variable with value zero for no sport at T0 and T1, or those who stopped doing sports from T0 to T1. We assigned value one for those who were doing sports at both T0 and T1 or those who started doing sports from T0 to T1.

SES was measured with one question regarding family finances. The pupils were asked about how “well off” they considered their family to be. The answers ranged from one (low family finances) to five (very good family finances). This question has been used to measure SES among adolescents in several studies [30]. All of the pupils reported their gender and school class.

The questions assessing attitude towards PE were designed to map attitudes towards physical activity among adolescents, and the same questions have been used in a national survey in 2011 which aimed to investigate PA habits among children and adolescents in Norway [31].

Perception of parental school support was assessed using the five-item HBSC parental support at school scale, which focuses on parental involvement and encouragement in school-related tasks and activities (presented in Table 1 and in the appendix) [32]. Items were measured on a 5-point Likert scale (1: strongly disagree; 5: strongly agree). Reliability and validity of the HBSC scale have been confirmed, and the scale has been used in multiple studies [32].

The variable “duration of active transportation to school” was based on the question “how do you normally travel to school?” Using a car or bus was assigned value zero, whereas walk and bicycle attained value one. This question was then multiplied with a question concerning the duration of the transport to school. This variable had values from 0 to 5 and was fairly normally distributed (skewness 0.33). We entered the active transportation measure as a continuous variable in the linear regression models. We will maintain that the five level categories with increasing active transport time can justify such a solution.

We summarized the number of other organized activities, excluding sports, at T0 and T1. These included cultural activities, playing in a school band, scouting, and congregational activities among other things. We computed a change variable by subtracting the number of activities at T0 from T1 called “change of nonsports activities.” This variable was only used as a predictor in the logistic regression analysis.

Self-rated health was assessed by the question: “how do you think your health is?” with the response alternatives “very good,” “good,” “not so good,” and “poor.” The number of categories was reduced, by combining “not so good” and “poor.” We recoded the variable into two dummy variables for the linear regression analyses, where the most negative response was the comparison category.

Body image/dissatisfaction was assessed by the question “what do you think about your body?” with the response alternatives “too thin,” “a bit too thin,” “normal,” “a bit too thick,” “too thick,” and “I do not think about it.” The number of categories was reduced to three, by combining “too thin” and “a bit too thin,” “too thick” and “a bit too thick,” and “normal” and “I do not think about it.” We recoded the variable into two dummies, where the most positive response (normal/do not think about it) was the comparison category [33].

### 2.2. Statistical Analyses

The scales were coded and recoded so that high values reflected increased levels of the variable in question. Items within a construct were also recoded to indicate the same direction. Cronbach's *α* was computed to estimate the internal consistency of all of the constructs, and values ranged from 0.70 to 0.89 (Table 1). The distributions of the scale variables were assessed with histograms and skewness. The variables were normally distributed except for parent support at T0 (skewness −1.75). In Table 1, descriptive statistics of frequencies, including percentages, means, and standard deviations, are presented for the categorical and continuous variables.

We presented the participation in sports activities, nonsports activities, and the students not in organized activities as numbers and percentages with 95% confidence intervals (Table 2).

In the predictor analyses, physical activity at T1 and persistent/started doing sports were the dependent variables. The independent variables were SES, peer support, parent support, attitude towards physical education, duration of active transport to school, change in nonsports activities during the two years, body dissatisfaction, and self-rated health.

We performed multiple linear regression analyses to explore which factors could predict level of physical activity at T1 (temporal causal analyses). Firstly, we explored the associations with one adjusting and predictor variable at a time. Secondly, we controlled for gender, age, and socioeconomic status (at T0) in adjusted analyses, entering each predictor one by one in the model. Finally, we did a full model analysis where adjusting variables and all the significant predictors from the adjusted analyses were entered in the model. We also performed a residual change analysis entering physical activity at T0 in the full model linear regression model (not shown in the table).

We used binary logistic regression analyses to explore which factors could predict who started or persisted doing sports from T0 to T1. We first explored the associations with one predictor at a time, before conducting adjusted analyses, correspondingly as for the linear regressions. Finally, we did a full model analysis where the adjusting variables and all the significant predictors from the adjusted analyses were entered in the model.

### 2.3. Ethics

The study was approved by the Norwegian regional committee for ethics in medical research, approval number “2011/510 REK vest.” Student participation was confidential and voluntary. Informed written consent was obtained from the parents and the students.

## 3. Results

Table 1 shows that most pupils were content with their family affluence. The majority had a rather passive transport to school. Most pupils reported good or very good self-rated health, and that they accepted their body shape or did not think about it.

Table 2 reveals that the youngest age group had high persistence in sports, both girls (63%) and boys (71%). Both genders reduced their participation in sports significantly from 8th to 10th grade: from 65% in 8th grade to 51% in 10th grade for girls and from 65% to 53% for boys, with CIs that did not overlap. Other leisure time activities than sports (nonsports activities) were quite stable for both girls and boys in this age group. The number of students who were not in organized leisure time activity increased significantly for both genders, from 24% in 8th grade to 39% in 10th grade for girls and from 24% to 37% for boys (CIs not overlapping).

Table 3 presents the results from the predictor analyses with PA as outcome. In the adjusted analyses, all predictors, except for the body dissatisfaction category “too thin,” had a significant impact on PA two years later. In the full model analysis, parent support, attitudes towards physical education, duration of active transport to school, and self-rated health (both good and very good) had a significant impact on PA two years later. The full model revealed that the explained variance was rather modest (0.08).

In the residual change analyses (not shown in the table), we entered physical activity at T0 in the linear regression model. The full model analysis revealed the following predictors as significant factors: age (B: −0.07, *p*: 0.02), PA at T0 (B: 0.34, *p* < 0.001), parent support at T0 (B: 0.06, *p* < 0.05), duration of active transport to school (B: 0.06, *p*: 0.04), and self-rated health (very good) (B: 0.12, *p*: 0.02).

Table 4 summarizes the results from the binary logistic regression analysis with the outcome “persistent and started doing sports.” In the adjusted analyses, all predictors, except for “duration of active transport to school” and the body dissatisfaction category “too thin,” were significantly associated with the outcome. The full model analysis revealed the following significant factors: age (OR: 0.58, *p* < 0.001), socioeconomic status (SES) (OR: 1.24, *p*: 0.03), increased nonsports activity during the two years (OR: 0.86, *p*: 0.03), and self-rated health (OR: 1.63, *p*: 0.03 and OR: 2.48, *p* < 0.001) for good and very good, respectively. The explained variance in the full model analysis was also for this model rather modest (0.09).

## 4. Discussion

We found a significant reduction in sports participation during early adolescence, most pronounced, from 13 to 15 years. Our finding that girls as well as boys in their early teens experienced a conspicuous reduction in sports participation, is in line with other studies [14, 26]. Factors which predicted being physically active after two years were a positive attitude towards physical education, perceived support from parents, if the student travelled to school in an active way (walk/bicycle) and also how the student rated his or her own health. The last three factors also predicted improvements of PA during the two years. The predictors of persisting or starting doing sports were increasing levels of self-rated health, increasing socioeconomic status, whereas increasing engagement in nonsports activities was associated with reduced participation in sports.

In the present study, we revealed that parent support was a significant factor of PA in youths. There are conflicting results from other papers regarding parent support and physical activity. In a metareview article from 2012, family support was identified as a correlate of PA in children and adolescents [34]. In a review of 46 articles examining change in PA in children and adolescents aged 4–18 years, they found that parental support was not associated with change in physical activity [35]. Similar findings were confirmed in a longitudinal study from the USA where they investigated predictors of PA among 578 adolescents between 10 and 16 years old. In this study, neither parent nor peer support towards PA were predictive of PA among boys or girls at follow-up [19]. In our study, parent support was a significant predictor of persisting or starting doing sports in the adjusted, but not in the full model analysis.

A Danish longitudinal school study from 2017 explored the extent to which parental involvement/role modelling had a beneficial impact in children's participation in organized sports. 1096 children/adolescents in the same age group as in the current study were followed, and the results suggest that parental involvement in children's sport increases the likelihood that the child will participate in organized sports [36]. However, not all parental involvement was beneficial for children's involvement in sports. The quality of the parental support may be important. In the current study, parent support was focused on parental involvement and encouragement in school-related tasks and activities. In spite our measure of parental support was not focused on PA or sports, we revealed that it was important for maintaining PA. The explanation is likely that parents supporting their children in school work are supportive also in leisure PA.

In our study, peer support was only a significant predictor of youth PA in the adjusted analysis, but not in the full model analysis. In a recent paper from Australia, peers influence children's PA most consistently through encouragement and positive modelling in sports activities [37]. Our peer support measure did not specifically pertain to sports.

With respect to SES, our study revealed that it was significantly associated with participation in sports. In the literature, the association between SES and PA is inconsistent [26]. In a Brazilian longitudinal school study, 4120 adolescents from 11 to 15 years were followed. SES did not predict PA change in girls while boys from low-income families improved their PA [38]. In most studies, however, young people from low-affluence families are less likely to be regularly active or participate in sports [17]. In two studies from the USA [39, 40] and two studies from Norway [18, 26], PA decline was more pronounced among those with lower family income and parental educational level.

A recent cohort study with objectively measured PA showed that self-efficacy and low perception of barriers to physical activity were important predictive factors for maintenance of PA in a similar age group as in our study [41]. Both self-efficacy and enjoyment were predictors of PA in a study from the US [19]. Our study revealed that a positive attitude towards physical education significantly predicted PA after two years, which is also in line with other studies [19]. We are fully aware that the concept “attitudes to PE” is not synonymous with self-efficacy. However, from the appendix, we can see that many of the statements express general expectancy and mastery beliefs that are related to self-efficacy. The present study and other studies have shown that level of physical activity at baseline is a strong predictor of level of physical activity at follow-up [18, 19]. It seems therefore important to foster mastering experiences, and positive attitude during physical education as health behaviours established early in life will influence lifestyle choices later.

The body dissatisfaction category “too thick” was significantly and negatively associated with both PA after two years and starting/persisting in doing sports. However, in the full model analyses, adjusting for the associations between the predictors, the impact from body dissatisfaction became statistically insignificant. A study among female undergraduates found that PA was associated with lower levels of body dissatisfaction [8], but the positive impact depended upon motivational factors. If the participants were motivated by weight and appearance intentions, the positive impact disappeared. This is in line with an earlier study including 2527 participants amongst tertiary school students from the same county in Norway as the present, proving an association between sports with leanness advantage and body dissatisfaction, especially among girls [9].

Increasing self-rated health predicted both PA and maintaining sports activity in the current study. The Norwegian study cited above revealed that increasing time spent on sports was related to improved self-rated health in a dose-response manner [9]. The present study supports a causal association. A former study revealed that this association is bidirectional [11]. A former Norwegian study pertained to other self-concepts than the present, revealing that also dissatisfaction with life was a predictor of diminished PA during adolescence [18].

A Swedish study showed that a school-based cognitive behavioural program for depression prevention positively influenced SRH over 12 months [42]. It is, therefore, tempting to suggest that improving self-worth and self-concepts may be a point of departure for maintaining and improving PA during adolescence. However, the present study only gives us observational evidence that improved SRH can result in maintenance and increase in PA and sports participation. We need experimental evidence that improved SRH can maintain and increase PA. However, the present study gives support for a causal link between SRH and PA/sports participation.

The strengths of the current study include its longitudinal design and that it was based on data from a large sample of adolescents, where the gender distribution and class distribution was quite similar. Additionally, we performed adjusted analyses and full model analyses in order to control for the relations between the predictors. We also performed residual change analysis in order to ascertain causality more firmly.

About 20% of nonparticipation in the study was due to absence from school or that students chose not to fill out the survey. It is likely that these students had less PA than the ones who participated in the study. This could have led to over-representation of PA in our sample, which may result in weaker effect sizes than in the whole study-population. The study was limited by the fact that the variables were measured at two time-points only. The explained variances for the two outcomes were rather modest, indicating that adolescent PA and sports participation depend on more factors than we have examined.

The data were collected using self-reporting, which may overestimate the associations investigated due to common method variance. Studies using self-reported measures usually find more physical activity than those using objective measures [43]. The survey pertained to a broad spectrum of health and health behaviour issues. Some of the predicting constructs were not focused on PA and sports participation, and comparison with other research may therefore be difficult. We are aware that other methods for ascertaining causal inference may be more valid than ours, such as cross-lagged analyses. However, we adjusted for relevant confounders and complemented the temporal causal method with residual change analyses.

We performed stratified analyses based on age and gender in order to evaluate interactions. Vastly overlapping CIs confirmed that we were able to analyse both genders and age groups together, using age and gender as adjusting variables.

## 5. Conclusion

Health promotion efforts aiming at increasing active school transportation, parental support, and subjective health seem important for maintenance of physical activity and sports participation during adolescence. Attitudes towards physical education may improve by adapting physical education to individual needs and interests and can function as an additional promotive factor.

## Figures and Tables

**Table 1:** Continuous and categorical outcome variables and predictors used in the analysis with Cronbach's alpha, mean value, standard deviation, and maximum variability. The items for these constructs are shown in the appendix.

Continuous variables: outcome and predictors	Response options (Likert scale)	Valid response (%)	Cronbach's alpha for the mean score	Mean (SD)	Range
Physical activity at T1	7 (1–7)	1183	0.70	4.34 (1.5)	0–7
Attitude towards physical education T0	5 (1–5)	1219	0.89	3.75 (0.85)	1–5
Parent support T0	5 (1–5)	1200	0.84	4.57 (0.57)	1–5
Peer support T0	5 (1–5)	1203	0.77	4.15 (0.62)	1–5
Duration of active transportation to school		1200 (97.9)		
Passive transport		509 (51)			
Active up to five minutes		179 (18)			
Active six to fifteen minutes		256 (25)			
Active sixteen to thirty minutes		57 (6)			
Active thirty-one to sixty minutes		5 (0.5)			
Socioeconomic status		1181 (96.4)		3.85 ^*∗*^(0.66)	
Categorical outcome and predictors		*N* (%)			
Persistent and started doing sports during two years		710 (58)			
Abstaining or quitting sports during two years		515 (42)			
Nonsports activities at T0					
No other activities		579 (47)			
One nonsports activity		420 (34)			
Two nonsports activities		162 (13)			
Three or more nonsports activities		64 (5.2)			
Self-rated health, poor		117 (9.6)			
Self-rated health, good		677 (55.3)			
Self-rated health, best		412 (33.6)			
Body dissatisfaction, too thick		314 (25.6)			
Body dissatisfaction, too thin		137 (11.2)			
Body dissatisfaction, normal/do not think about it		734 (59.9)			

^*∗*^The answers ranged from one (low family finances) to five (very good family finances).

**Table 2:** Number of the adolescents who are doing sports and other leisure time activities (nonsports activity) or are not in organized activity (neither sports nor other leisure time activities) at T0 and T1. Stratified for gender and grade level with percentages and 95% confidence intervals.

Grade level and sex (N)	T0			T1		
	Sports/exercise, per cent (95% CI) (N)	Nonsports activity, per cent (95% CI) (N)	Not in organized activity, per cent (95% CI) (N)	Sports/exercise, per cent (95% CI) (N)	Nonsports activity, per cent (95% CI) (N)	Not in organized activity, per cent (95% CI) (N)
6th grade ⟶ 8th grade, female (233)	63 (56–69) (146)	22 (17–28) (52)	15 (11–20) (35)	68 (62–73) (158)	12 (8–17) (29)	20 (15–25) (46)
6th grade ⟶ 8th grade, male (242)	71 (66–77) (173)	12 (8–17) (29)	17 (12–22) (40)	65 (59–71) (159)	8 (5–12) (19)	27 (21–32) (64)
8th grade ⟶ 10th grade, female (377)	65 (60–70) (246)	11 (8–14) (41)	24 (20–28) (90)	51 (46–56) (193)	10 (7–13) (36)	39 (34–44) (148)
8th grade ⟶ 10th grade, male (367)	65 (60–70) (240)	11 (8–14) (39)	24 (20–29) (88)	53 (48–58) (195)	10 (7–13) (35)	37 (33–42) (137)

**Table 3:** Linear regression analyses, unadjusted and adjusted for gender, age, and socioeconomic status, and full model analysis with the outcome variable physical activity mean at T1.

Variable	Unadjusted, unstandardized regression coefficients, 95% CIs and *p* values			Adjusted^*∗*^, unstandardized regression coefficients, 95% CIs and *p* values			Full model, unstandardized regression coefficients, 95% CIs and *p* values		
	B	CI	*p*	B	CI	*p*	B	CI	*p*
Gender	−0.088	(−0.43; −0.092)	0.003				−0.056	(−0.34; 0.005)	0.06
Age	−0.075	(−0.41; −0.056)	0.010				−0.04	(−0.3; 0.06)	0.18
Socioeconomic status	0.068	(0.023; 0.28)	0.022				0.01	(-0.11; 0.16)	0.71
Peer support T0	0.12	(0.14; 0.42)	0.001	0.1	(0.1; 0.38)	0.001	0.018	(−0.10; 0.19)	0.56
Parent support T0	0.13	(0.19; 0.49)	0.001	0.12	(0.17; 0.48)	0.001	0.072	(0.03; 0.35)	0.02
Attitudes towards gymnastics T0	0.15	(0.17; 0.37)	0.001	0.13	(0.14; 0.34)	0.001	0.068	(0.01; 0.23)	0.03
Duration of active transport to school	0.084	(0.036; 0.20)	0.004	0.08	(0.03; 0.19)	0.006	0.08	(0.03; 0.19)	0.006
Body image: too thick T0	−0.12	(−0.59; −0.19)	0.001	−0.09	(−0.51; −0.09)	0.005	−0.01	(−0.26; 0.17)	0.66
Body image: too thin T0	−0.030	(−0.42; 0.14)	0.32	−0.03	(−0.43; 0.13)	0.28			
SRH best	0.35	(0.79; 1.40)	0.001	0.32	(0.71; 1.34)	0.001	0.26	(0.49; 1.17)	0.001
SRH good	0.15	(0.15; 0.74)	0.003	0.14	(0.13; 0.72)	0.005	0.11	(0.01; 0.62)	0.04
Explained variance									0.08

^*∗*^Adjusted for age, gender, and socioeconomic status.

**Table 4:** Logistic regression analyses, unadjusted and adjusted for gender, age, and socioeconomic status, and full model analysis with the outcome variable persistent and started doing sports.

Variable	Unadjusted, odds ratios, 95% CIs and *p* values			Adjusted^*∗*^, odds ratios, 95% CIs and *p* values			Full model, odds ratios, 95% CIs and *p* values		
	OR	CI	*p*	OR	CI	*p*	OR	CI	*p*
Gender	0.97	(0.77; 1.22)	0.82				1.07	(0.83; 1.39)	0.61
Age	0.55	(0.43; 0.7)	0.001				0.58	(0.45; 0.76)	0.001
Socioeconomic status	1.39	(1.17; 1.66)	0.001				1.24	(1.02; 1.51)	0.03
Peer support T0	1.41	(1.17; 1.7)	0.001	1.29	(1.06; 1.57)	0.012	1.09	(0.88; 1.36)	0.43
Parent support T0	1.44	(1.18; 1.76)	0.001	1.33	(1.08; 1.65)	0.008	1.14	(0.9; 1.44)	0.28
Attitudes towards gymnastics T0	1.33	(1.16; 1.52)	0.001	1.24	(1.08; 1.43)	0.003	1.09	(0.93; 1.28)	0.29
Duration of active transport to school	1.06	(0.96; 1.18)	0.25	1.05	(0.94; 1.17)	0.39			
Change to other nonsports activities during the 2 years	0.85	(0.75; 0.97)	0.014	0.85	(0.75; 0.98)	0.02	0.86	(0.75; 0.99)	0.03
SRH good	2.1	(1.4; 3.15)	0.001	1.96	(1.3; 2.98)	0.001	1.63	(1.05; 2.54)	0.03
SRH best	3.91	(2.54; 6.01)	0.001	3.27	(2.1; 5.11)	0.001	2.48	(1.51; 4.06)	0.001
Body image: too thick T0	0.57	(0.44; 0.75)	0.001	0.64	(0.48; 0.84)	0.002	0.78	(0.57; 1.06)	0.11
Body image: too thin T0	0.89	(0.62; 1.29)	0.55	0.84	(0.57; 1.23)	0.37			
Explained variance									0.09

^*∗*^Adjusted for age, gender, and SES.

## Data Availability

The datasets used and analysed during the current study are available from the corresponding author on request.

## Appendix

  Items used for the construction of composite scores for physical activity, attitude towards physical education, peer support, and parent support measured at T0.

### A. Physical Activity

  Outside school hours: how many days a week do you play sports or exercise so that you get out of breath or sweat?
  During the last seven days, how many of these days have you been physically active for at least 60 minutes?

### B. Attitude towards Physical Education

  Physical education helps me feel more secure on my own body
  Physical education help making me enjoy being physically active
  Physical education teaches me how the body works
  Physical education teaches me how I should train to get in better shape
  Physical education teaches me what good health is
  Physical education teaches me how to be better at sports
  Physical education helps me understand that my body is well suited for being physically active
  Physical education helps me understand that I can be proud of my own body
  Physical education helps me understand that I am a well-functioning human being

### C. Parent Support

  If I have a problem at school, my parents will be there to help me
  My parents are willing to attend meetings with my teachers at school
  My parents encourage me to do well in school
  My parents are interested in what happens to me at school
  My parents are willing to help me with my school work

### D. Peer Support

  The students in my class enjoy being together
  Most of the students in my class are nice and helpful
  My fellow students accept me as I am
